# Inflammatory Blood Parameters as Biomarkers for Response to Immune Checkpoint Inhibition in Metastatic Melanoma Patients

**DOI:** 10.3390/biomedicines10092135

**Published:** 2022-08-31

**Authors:** Ken Kudura, Lukas Nussbaumer, Robert Foerster, Lucas Basler

**Affiliations:** 1 Department of Nuclear Medicine, University Hospital Zurich, 8006 Zurich, Switzerland; 2 Faculty of Medicine, University of Zurich, 8091 Zurich, Switzerland; 3 Institute of Radiooncology, Cantonal Hospital Winterthur, 8400 Winterthur, Switzerland; 4 Institute of Radiooncology, Cantonal Hospital Aarau, 5001 Aarau, Switzerland

**Keywords:** positron emission tomography/computed tomography, melanoma, immunotherapy, CTLA-4, PD-1, blood biomarkers, outcome prediction

## Abstract

**Simple Summary:**

Immune checkpoint inhibitors (ICIs) have become a pillar of advanced melanoma treatment. Given the moderate response rate to ICIs in metastatic melanoma patients and the potentially severe toxicity of ICIs, the distinction between nonresponders and responders is crucial and challenging at the same time. Several biomarkers of response to immune checkpoint inhibition have been discussed in recent studies with conflicting results, and are so far not implemented in clinical routine. In this context, the validation of biomarkers obtained easily in clinical practice and predicting ICIs’ efficacy could improve the response rate and prevent nonresponders from immunotoxicity. Here, we provide evidence from a large cohort of metastatic melanoma patients that inflammatory blood parameters predicted the short- and long-term responses to ICIs with strong prediction power. Our results suggested the validation of inflammatory blood parameters as biomarkers predicting immunotherapies’ efficacity in metastatic melanoma patients. However, confounding factors interfering with myelopoiesis should also be taken into consideration.

**Abstract:**

**Objectives:** We aimed to investigate whether inflammatory parameters in peripheral blood at baseline and during the first six months of treatment could predict the short- and long-term outcomes of metastatic melanoma patients treated with immune checkpoint inhibitors (ICIs). **Methods:** This single-center retrospective study considered patients with metastatic melanoma treated with either single or dual checkpoint inhibition. Blood sample tests were scheduled together with ^18^F-2-fluor-2-desoxy-D-glucose positron emission tomography/computed tomography (FDG-PET/CT) scans at baseline and at three and six months after initiation of ICI treatment. The short-term response to ICIs was assessed using FDG-PET/CT scans. The long-term response to ICIs was assessed using the overall survival OS and progression-free survival PFS as endpoints. **Results:** A total of 100 patients with metastatic melanoma were included (female, *n* = 31; male, *n* = 69). The median age was 68 years (interquartile range (IQR): 53–74 years). A total of 82% of the cohort displayed a disease control (DC), while 18% presented a progressive disease (PD) after six months of ICIs. Patients with DC after six months of ICIs showed a lower median of the neutrophils-to-lymphocytes ratio (NLR) toward patients with PD, with no significant prediction power of NLR neither in the short nor in the long term. The count of neutrophils at the baseline time point (TP 0) (*p* = 0.037) and erythrocytes three months after treatment start (TP 1) (*p* = 0.010) were strong predictive parameters of a DC six months after treatment start. Erythrocytes (*p* < 0.001) and lymphocytes (*p* = 0.021) were strong biomarkers predictive of a favorable OS. Erythrocytes (*p* = 0.013) and lymphocytes (*p* = 0.017) also showed a significant prediction power for a favorable PFS. **Conclusions:** Inflammatory blood parameters predicted the short- and long-term response to ICIs with a strong predictive power. Our results suggested the validation of inflammatory blood parameters as biomarkers that predict immunotherapies’ efficacity in metastatic melanoma patients. However, confounding factors that interfere with myelopoiesis should also be taken into consideration.

## 1. Introduction

Metastatic melanoma was previously a disease with limited treatment options and a poor prognosis [1]. Metastatic melanoma was the first malignant tumor to be successfully treated with immune checkpoint inhibition [2]. Monoclonal antibodies targeting cytotoxic T-lymphocyte-associated antigen-4 (CTLA-4), programmed cell death protein-1 (PD-1), and PD-ligand 1 (PD-L1), which are defined as immune checkpoint inhibitors (ICIs), have significantly improved the outcome of metastatic melanoma patients and so have become a pillar of advanced melanoma treatment [1,3]. However, a durable response is only seen in a small proportion of treated patients (about 40–50%) [2], while up to 60% of all treated patients are at risk for potentially severe immune-related adverse events [1,2,3,4]. In order to address these challenges, there has been increasing effort toward identifying biomarkers that differentiate responders from nonresponders. Several biomarkers of response to immune checkpoint inhibition have been discussed [2,4,5,6,7,8,9,10,11,12].

In a systematic review published in 2015, Petrelli et al. investigated the prognostic value of lactate dehydrogenase (LDH) in solid tumors. High LDH levels were associated with poor survival in solid tumors, particularly in melanoma [9]. Recent investigations confirmed the prognostic value of LDH in metastatic melanoma patients treated with ICI, implying that high LDH levels at baseline were a poor prognostic factor [10].

The immunohistochemical expression of CTLA-4, PD-1, and PD-L1 in tumor-infiltrating lymphocytes has also been evaluated as a predictive marker of response to ICIs in melanoma patients with controversial results. Recent studies suggested that the immunohistochemical expression of tumor-infiltrating lymphocytes might not be an adequate biomarker of response to ICIs in melanoma patients, since patients with a lack of expression of inhibitory checkpoint proteins also do not respond to ICIs [6,11].

Cutaneous melanoma also displays a high mutation burden, although not all melanoma types exhibit a high mutation burden. The tumor mutation burden (TMB) as a biomarker of response to ICIs in melanoma patients has also been discussed in the recent literature, with conflicting results. It was not clarified whether a high tumor burden could be used as predictor of response to ICIs [2,7,8,12].

However, while these biological features have been described as predictive biomarkers in various studies, they are far from being evaluated routinely. Therefore, predictive biomarkers with clinically convenient, practical, and cost-effective screening are needed [13].

Due to the mechanism of action of ICIs, it has become evident that the systemic inflammatory response to treatment, such as a decreased or increased myelopoiesis, plays a crucial role in disease progression, and therefore in the clinical outcome after treatment with ICIs. Systemic inflammation exhibits alterations in peripheral blood [14].

In light of this knowledge, we aimed to investigate whether inflammatory parameters in peripheral blood at baseline and in the early stage of treatment with ICIs can predict the short- and long-term outcomes of metastatic melanoma patients treated with immune checkpoint inhibition.

## 2. Methods

### 2.1. Patient Cohort

This single-center retrospective study was conducted using a research ethics board-approved protocol (KEK-ZH-Nr: 2014-0193) in compliance with Good Clinical Practice (GCP) rules and the Declaration of Helsinki.

Patients were eligible for inclusion if they had a histopathologically confirmed metastatic melanoma treated with either single checkpoint inhibition (anti-PD-1) or dual checkpoint inhibition (anti-PD-1/anti-CTLA-4) between 2013 and 2019 at the Department of Dermatology of the University Hospital Zurich in Switzerland.

All included patients consented the use of their clinical data for research purposes.

### 2.2. Patient Characteristics

Patient characteristics (such as age, sex, American Joint Committee on Cancer (AJCC) stage, histopathology of primary tumor, anatomical site of primary tumor, anatomical site of metastases, prior treatment, immunotherapy agent, date of treatment start, and clinical follow-up from treatment start to date of disease progression or patient death) were provided based on internal clinical records.

### 2.3. Blood Parameters

Blood sample tests taken in clinical routine at regular intervals were mandatory for inclusion. Since the blood sample tests were scheduled together with the ^18^F-2-fluor-2-desoxy-D-glucose positron emission tomography/computed tomography (FDG-PET/CT) scans performed at baseline and for treatment response assessment during the first six months after initiation of ICI treatment, three time points (TPs) were considered for the blood sample tests: at baseline (TP 0), three months after initiation of ICIs (TP 1), and six months after initiation of ICIs (TP 2). The average interval between blood samples was 107.1 days between TP 0 and TP 1 and 93.5 days between TP 1 and TP 2.

The count of following proteins and cells in peripheral blood was recorded at all three time points (TP 0, TP 1, and TP 2) per patient based on internal clinical records: basophiles (g/L), c-reactive protein (mg/L), erythrocytes (per pL), leucocytes (g/L), lymphocytes (g/L), monocytes (g/L), neutrophils (g/L), and thrombocytes (g/L). The neutrophils-to-lymphocytes-ratio (NLR) was then calculated by dividing the absolute neutrophils count by the absolute lymphocytes count.

### 2.4. Clinical Outcome Short-Term Response to Immune Checkpoint Inhibition

The short-term response to ICIs was assessed using FDG-PET/CT scans performed in clinical routine six months after treatment start.

All considered FDG-PET/CT scans were performed in clinical routine at the Department of Nuclear Medicine of the University Hospital Zurich according to the department’s standard protocol.

Most of the examinations were performed from the vertex of the skull to the thighs in a supine position. Only if the primary melanoma was located in the lower extremities, whole-body FDG-PET/CT scans were performed. For attenuation correction, a CT scan without contrast medium was performed (slice thickness 3.75 mm; field of view 50 cm; matrix size 512 × 512; tube potential 120 kV; tube current modulation between 15 and 80 mA) immediately followed by the PET acquisition (matrix size 256 × 256; field of view 70 cm) using the time-of-flight (TOF) technique.

Image acquisition began 60 min after the administration of a body mass index (BMI)-adapted ^18^F-FDG dose and a blood glucose level below 160 mg/dl at the time of ^18^F-FDG injection. Patients were asked to fast at least 4 h prior the intravenous ^18^F-FDG administration.

Brain metastases were not included due to the surrounding physiological FDG uptake of the cerebral cortex, as well as the difficult morphological assessment of native CT scans.

Treatment response was assessed according to RECIST 1.1.

Based on their short-term outcome six months after initiation of ICIs, patients were dichotomized into two groups: patients with disease control (CR, PR, SD) and patients with disease progression (PD).

In order to predict the dichotomized response six months after ICIs based on FDG-PET/CT scans (disease control vs. no disease control), the inflammatory blood parameters at TP 0 and TP 1 were collected and further analyzed.

### 2.5. Clinical Outcome Long-Term Response to Immune Checkpoint Inhibition

The long-term response to ICIs was assessed using the overall survival (OS) and progression-free survival (PFS) as endpoints. The overall survival was defined as the time from first treatment to death or last follow-up, while the progression-free survival was defined as the time from first treatment to disease progression or death.

In order to predict the long-term response to ICIs based on OS and PFS as endpoints, the inflammatory blood parameters at TP 0, TP 1, and TP 2 were collected and further analyzed.

### 2.6. Statistical Analysis

Statistical analysis was performed in R (version 3.3.3) by the R core team. Continuous variables such as blood parameters were summarized as median and range, and categorical variables as frequencies. For the prediction of the short-term response to ICIs, a stepwise binominal logistic regression (backward selection) was used. ROC analyses were performed to determine the optimal cut-off values of the significant blood parameters for the prediction of the short-term outcome. Regarding the prediction of the long-term response to ICIs, a regression analysis with time-varying covariates using a Cox proportional hazard model was performed. Statistical significance was accepted at *p* < 0.050.

## 3. Results

### 3.1. Patient Characteristics

A total of 100 patients with metastatic melanoma were included (female, *n* = 31; male, *n* = 69). The median age was 68 years (interquartile range (IQR): 53–74 years). The vast majority (*n* = 95) of the considered population was staged AJCC IV with a primary tumor mostly located in the head and neck, upper extremity, or body trunk. A total of 58% of the population had more than three melanoma metastases. In total, 670 melanoma metastases were detected on the FDG-PET/CT scans before initiation of ICIs, most frequently located in soft tissues, followed by lung and liver/spleen. A total of 84% of all patients were treated with single checkpoint inhibition, while 16% with were treated with double checkpoint inhibition. A total of 76% of all patients were pretreated at TP 0, while 24% did not receive any prior treatment (Table 1). 

### 3.2. Short-Term Response to Immune Checkpoint Inhibition

The short-term response to ICIs was assessed using FDG-PET/CT scans performed six months after initiation of treatment. A total of 8% showed a complete response, 40% a partial response, 34% a stable disease, and 18% a progressive disease.

**Figure 1:**
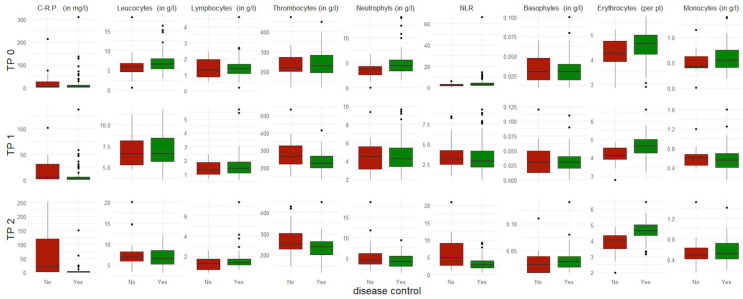
Boxplots of all included blood parameters per time point (TP) (at baseline TP 0 and at three and six months after starting immune checkpoint inhibition, respectively designated as TP 1 and TP 2) in patients with disease control at six months (displayed in green) versus patients with progressive disease at the same time (displayed in red).

In total, 82% of the cohort displayed a disease control, while 18% presented a progressive disease after six months of ICIs (Figure 1). 

An interesting dynamic in the NLR was observed over time in the first six months of therapy. While no important differences between both patients groups were seen at TP 0 and TP 1, a considerably lower median value of the NLR with a considerably narrower IQR was observed in patients with disease control compared to patients with progressive disease six months after ICIs (Table 2 and Table 3).

Our results showed a similar dynamic regarding c-reactive protein over time in the first six months of therapy, with an even lower median value and an even narrower IQR in patients with disease control compared to patients with progressive disease six months after ICIs (Table 2 and Table 3).

**Figure 2:**
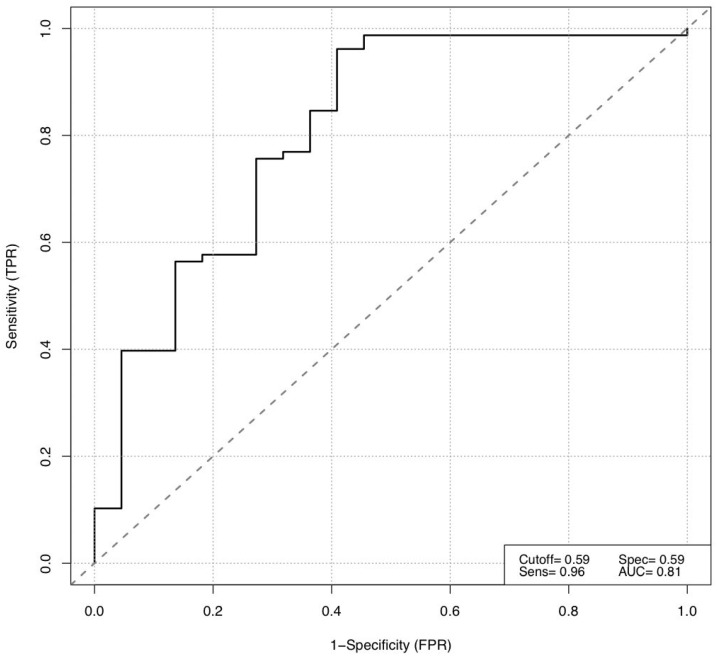
Receiver operating characteristic (ROC) of our binominal backward regression model (logit model) for short-term response prediction in metastatic melanoma patients treated for six months with immune checkpoint inhibition.

**Figure 3:**
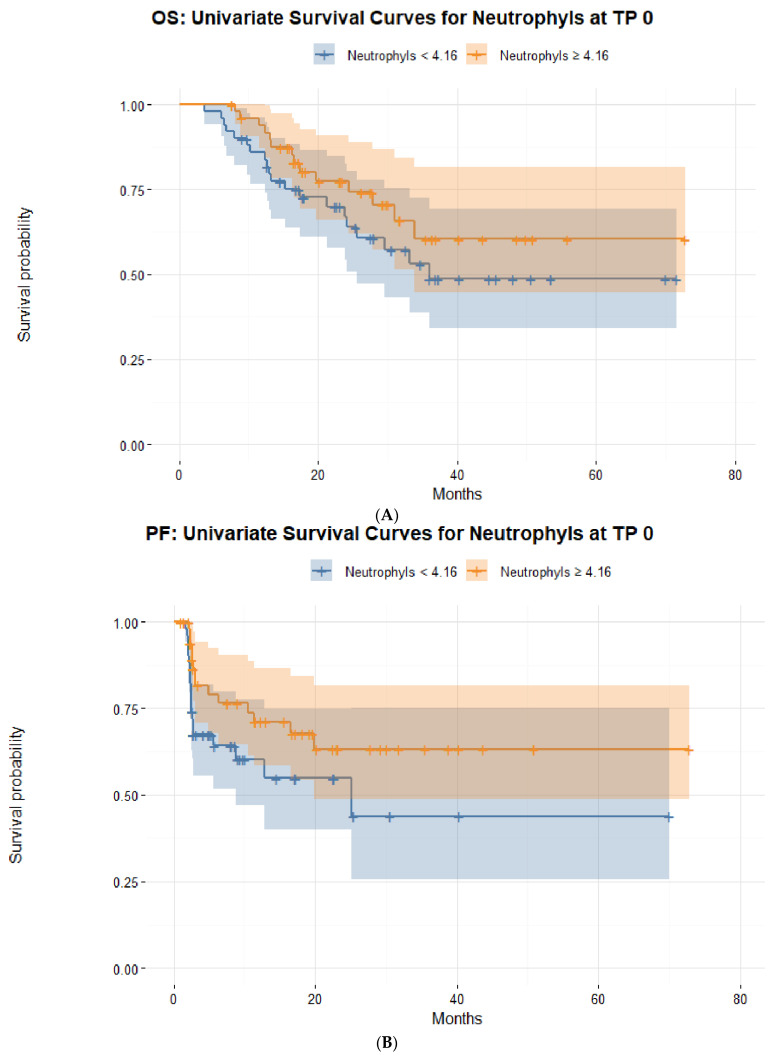
Kaplan–Meier survival curves stratified by the optimal cut-off count for neutrophils (g/L) at baseline in metastatic melanoma patients based on (**A**) overall survival and (**B**) progression-free survival.

**Figure 4:**
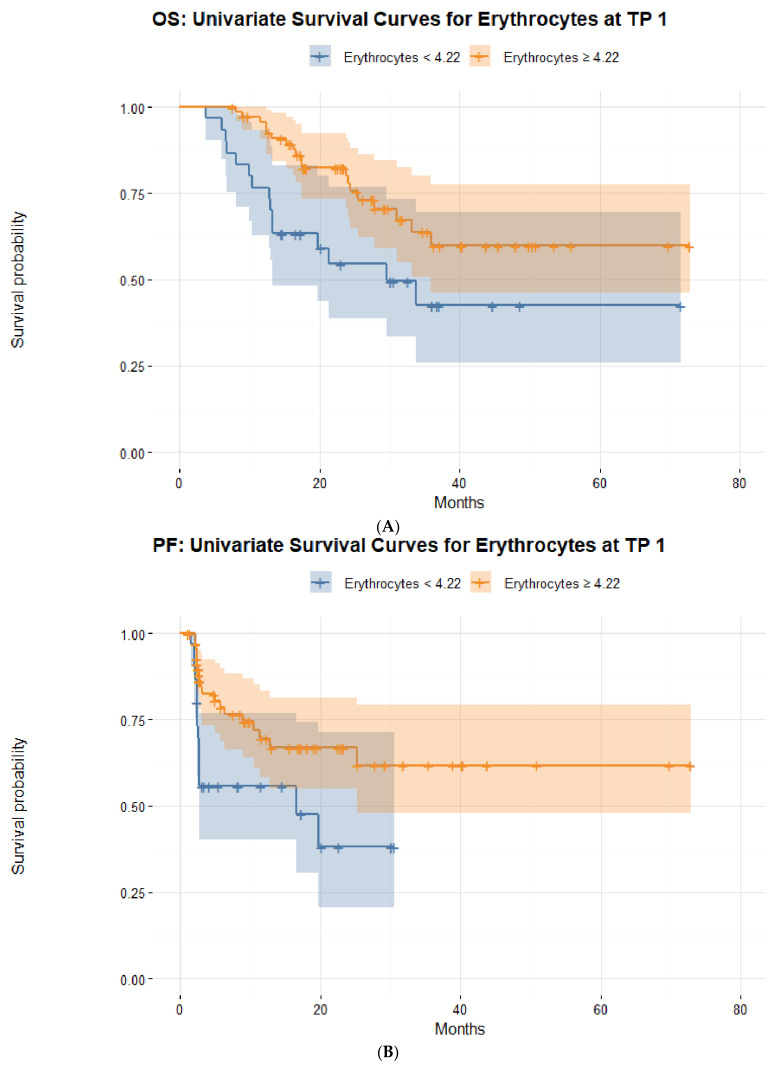
Kaplan–Meier survival curves stratified by the optimal cut-off count of erythrocytes (per pL) three months after initiation of immune checkpoint inhibition in metastatic melanoma patients based on (**A**) overall survival and (**B**) progression-free survival.

In order to predict the short-term response to ICIs at TP 2, a stepwise binominal logistic regression (backward selection) was performed using all collected blood parameters at TP 0 and TP 1. Among these blood parameters, the counts of neutrophils at TP 0 (*p* = 0.037) and erythrocytes at TP 1 (*p* = 0.010) were strong predictive parameters of a short-term response to ICIs. The performance of our binominal backward logistic regression model was assessed using the Akaike information criterion (AIC), resulting in an estimated low value of 89.7, which suggested a low estimated prediction error of the presented statistical model. For this purpose, the receiver operating characteristic (ROC) was also used, displaying a sensitivity (TPR) of 0.96, a specificity (FTR) of 0.59, and an area under the curve (AUC) of 0.81. The AUC was then used to define the optimal cut-off values for the two prognostic blood markers for short-term response to ICIs: neutrophils at TP 0 (4.1 g/L) and erythrocytes at TP 1 (4.22 per pL) (Figure 2, Figure 3 and Figure 4). 

### 3.3. Long-Term Response to Immune Checkpoint Inhibition

The long-term response to ICIs was assessed using the overall survival (OS) and progression-free survival (PFS) as endpoints.

The median follow up was 25.2 months (IQR 14.4–32.8), the median overall survival was 24.8 months (IQR 14.0–32.3), and median progression-free survival was 11.8 months (IQR 2.0–17.0). The mortality rate one year and two years after initiation of ICIs was 15% and 45%, respectively.

In order to predict the long-term response to ICIs based on OS and PFS as endpoints, patient characteristics such as age, sex, and type of ICIs, as well as all blood parameters collected at TP 0, TP 1, and TP 2, were further analyzed. Therefore, a regression analysis with time-varying covariates using a Cox proportional hazard model was performed to predict the long-term response to ICIs.

**Figure 5:**
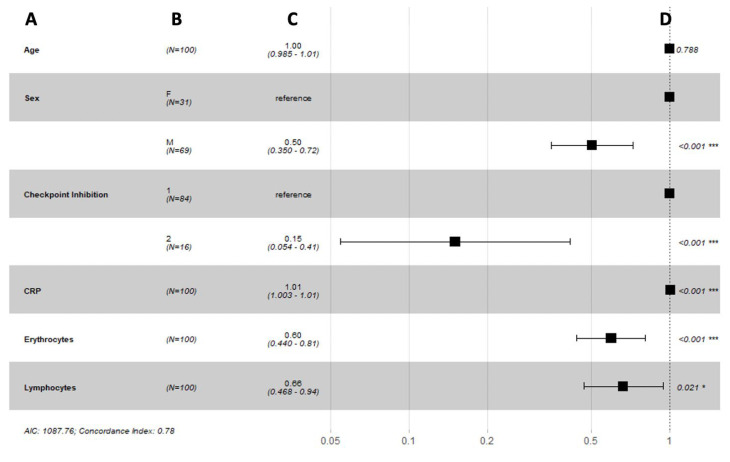
Forest plots summarizing the results of a Cox proportional hazard model analysis of overall survival with time-varying covariates: (**A**) variable; (**B**) number of patients; (**C**) hazard ratio (95% CI); (**D**) *p*-value. Abbreviations: F = female, M = male, 1 = single ICI, 2 = double ICI, CRP = c-reactive protein, AIC = Akaike information criterion. * and *** indicate significance at the 0.1 and <0.01 levels respectively.

Our long-term prediction model of the overall survival showed a good prediction power with a concordance index of 0.78 and suggested that sex (*p* < 0.001), ICI agent (*p* < 0.001), and blood parameters such as c-reactive protein (*p* < 0.001), erythrocytes (*p* < 0.001), and lymphocytes (*p* = 0.021) were strong biomarkers that were predictive of the overall survival in metastatic melanoma patients (Figure 5). 

**Figure 6:**
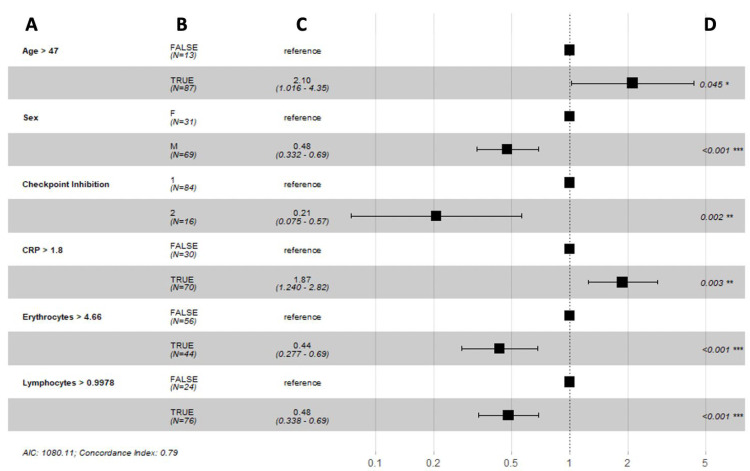
Forest plots summarizing the results of a Cox proportional hazard model analysis of overall survival with optimal cut-off values of time-varying covariates: (**A**) variable; (**B**) number of patients; (**C**) hazard ratio (95% CI); (**D**) *p*-value. Abbreviations: F = female, M = male, 1 = single ICI, 2 = double ICI, CRP = c-reactive protein, AIC = Akaike information criterion. *, ** and *** indicate significance at the 0.1, 0.05 and <0.01 levels respectively.

Furthermore, optimal cut-off values for these predictive biomarkers were defined as the values for which the two considered reference groups differed the most in their probability of survival over time. Male sex (hazard ratio (HR) 0.48, 95% confidence interval (CI) 0.33–0.69, *p* < 0.001), double checkpoint inhibition (HR 0.21, 95% CI 0.07–0.57, *p* = 0.002), erythrocytes > 4.66 per pL (HR 0.44, 95% CI 0.28–0.69, *p* < 0.001), and lymphocytes > 0.998 g/L (HR 0.48, 95% CI 0.34–0.69, *p* < 0.001) were associated with a longer overall survival. However, c-reactive protein > 1.80 (HR 1.87, 95% CI 1.24–2.82, *p* = 0.003) was associated with a poorer overall survival (Figure 6). 

**Figure 7:**
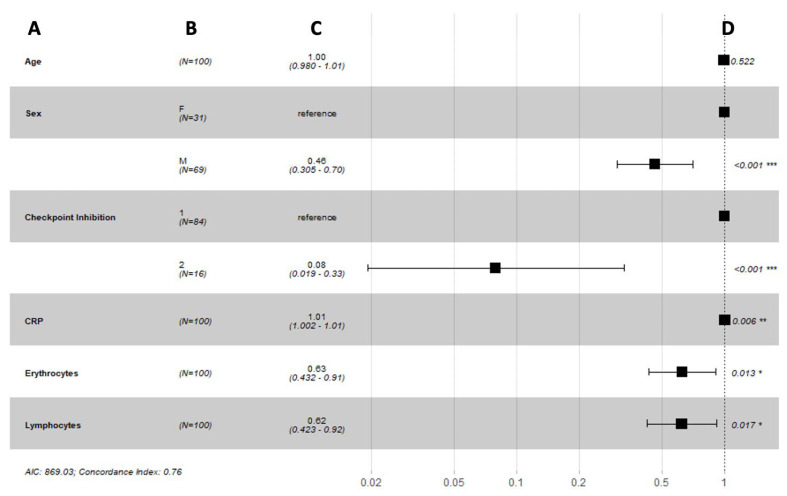
Forest plots summarizing the results of a Cox proportional hazard model analysis of progression-free survival with time-varying covariates: (**A**) variable; (**B**) number of patients; (**C**) hazard ratio (95% CI); (**D**) *p*-value. Abbreviations: F = female, M = male, 1 = single ICI, 2 = double ICI, CRP = c-reactive protein, AIC = Akaike information criterion. *, ** and *** indicate significance at the 0.1, 0.05 and <0.01 levels respectively.

Our long-term prediction model of the progression-free survival showed a good prediction power with a concordance index of 0.76 and suggested that sex (*p* < 0.001), ICI agent (*p* < 0.001), and blood parameters such as c-reactive protein (*p* = 0.006), erythrocytes (*p* = 0.013), and lymphocytes (*p* = 0.017) were strong biomarkers that were predictive of the progression-free survival in metastatic melanoma patients (Figure 7). 

**Figure 8:**
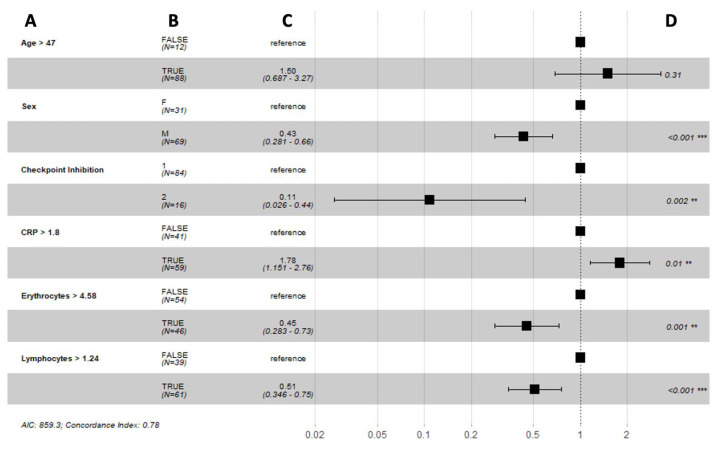
Forest plots summarizing the results of a Cox proportional hazard model analysis of progression-free survival with optimal cut-off values of time-varying covariates: (**A**) variable; (**B**) number of patients; (**C**) hazard ratio (95% CI); (**D**) *p*-value. Abbreviations: F = female, M = male, 1 = single ICI, 2 = double ICI, CRP = c-reactive protein, AIC = Akaike information criterion. ** and *** indicate significance at the 0.05 and <0.01 levels respectively.

Furthermore, optimal cut-off values for these predictive biomarkers were defined as the values for which the two considered reference groups differed the most in their probability of survival over time. Male sex (HR 0.45, 95% CI 0.29–0.68, *p* < 0.001), double checkpoint inhibition (HR 0.11, 95% CI 0.03–0.45, *p* = 0.002), erythrocytes > 4.66 (HR 0.44, 95% CI 0.28–0.70, *p* < 0.001), and lymphocytes > 0.998 (HR 0.52, 95% CI 0.35–0.76, *p* < 0.001) were associated with a longer progression-free survival. However, c-reactive protein > 1.79 (HR 1.79, 95% CI 1.15–2.77, *p* = 0.010) was associated with a poorer progression-free survival (Figure 8). 

## 4. Discussion

Immune checkpoint inhibitors have become a pillar of advanced melanoma treatment [1,5]. Given the moderate response rate to ICIs in metastatic melanoma patients and the potentially severe toxicity of ICIs, the distinction between nonresponders and responders is crucial and challenging at the same time. Several biomarkers of response to immune checkpoint inhibition have been discussed in recent studies with conflicting results, and are so far not implemented in clinical routine [2,4,5,6,7,8,9,10,11,12].

In this context, the validation of biomarkers obtained easily in clinical practice and predicting ICIs’ efficacy could improve the response rate and prevent nonresponders from immunotoxicity [15].

The mechanism of action of ICIs relies on an increased function of lymphocytes as natural killer (NK) cells and CD8+ cytotoxic T cells by modulating immune checkpoint proteins. NK cells destroy cells lacking major histocompatibility complex class I (MHC-1) as first line of defense against the tumor, while CD8+ cytotoxic T cells destroy tumor cells by releasing granula [16]. In light of this knowledge, we investigated whether higher levels of lymphocytes were correlated with improved outcomes in metastatic melanoma. Our results suggested that higher levels of lymphocytes in the first six months of treatment with ICIs were strongly associated with a better response in long term, both overall survival (with a cut-off value set at 1.00 g/L) and progression-free survival (with a cut-off value set at 1.24 g/L) in metastatic melanoma patients. Conversely, a lymphocytopenia during the first six months of treatment was associated with poor overall survival and progression-free survival.

Neutrophils can suppress T-cell proliferation or induce T-cell apoptosis and thus favor tumorigenesis, although neutrophils with a suppressive phenotype (i.e., against tumorigenesis and proliferation) can also be released from the bone marrow [16]. Interestingly, lower levels of neutrophils in the first six months of treatment with ICIs were not associated with a better outcome in our population, neither in the short nor in the long term. On the contrary, our results surprisingly showed that higher levels of neutrophils before treatment with ICIs (cut-off set at 4.16 g/L) were associated with better disease control six months after initiation of ICIs, suggesting that the initial count of neutrophils might play a more significant role in the first months of treatment than in the long term.

In light of the central role played by T cells in the destruction of tumor cells and neutrophils’ modulation of T cells’ function, the neutrophils-to-lymphocytes ratio (NLR) has drawn attention as a biomarker for response in the context of melanoma [16]. In our cohort, the group of patients with disease control after six months of ICIs showed a lower median of NLR compared to the group with progressive disease, although no significant predictive power of NLR was observed in the short or long-term.

Various recent investigations reported the NLR as a potential biomarker for response in melanoma. However, the following differences compared to our study design can be highlighted.

Cohen et al. analyzed recent investigations on the prognostic value of NLR and concluded that a high NLR was correlated with worse overall and disease-free survival. While their investigations included high-risk localized melanoma and metastatic melanoma treated with ICIs, targeted therapy, and metastasectomy, we focused on metastatic melanoma treated with ICIs only. In addition, one of the included studies with a single ICI (ipilimumab) considered 197 patients with unresectable stage III or stage IV melanoma and NLR measured at baseline and at three, six, and nine weeks after initiation of treatment, while the NLR in our investigation was assessed at baseline and at three and six months after initiating ICIs [16].

The last highlighted difference might emphasize the importance of the time point for the assessment of NLR, which has not been defined yet in the case of metastatic melanoma treated with immunotherapy.

Sacdalan et al. performed a meta-analysis on the utility of baseline NLR across several malignancies treated with ICIs, including melanoma. They reported that a high baseline NLR was associated with poorer outcomes [17]. Criscitiello et al. came to the same conclusion using the baseline NLR across solid tumors treated with ICIs [15]. Viñal et al. assessed the NLR at baseline and before the second dose of immunotherapy, but also the NLR trend in order to predict the response to treatment in advanced cancer patients treated with ICIs. They reported that the NLR at all three time points was a prognostic factor for survival [18].

Our results brought further innovative insights to light. Higher counts of erythrocytes in the first six months of treatment were associated with a better overall survival (>4.6/pL) and progression-free survival (>4.58/pL). Higher levels of erythrocytes after initiation of ICIs (>4.22/pL) were also associated with a better disease control in the short-term. Conversely, anemia was associated with poorer outcomes in short- and long-term.

The use of these inflammatory parameters in peripheral blood as predictive factors, particularly the use of neutrophils and the NLR, can be limited by processes that result in an increased number of circulating neutrophils, such as acute infections [6]. Interestingly, our results brought to light that higher levels of c-reactive protein during the first months of treatment were associated with a poorer outcome in the long term. In addition, the median counts of c-reactive protein during the first six months of treatment were higher in patients with progressive disease compared to patients with disease control six months after initiation of ICIs.

Finally, clinical parameters such as male sex and the use of a double agent for ICIs were also strong predictors of a favorable response to treatment.

One important limitation of our investigations should be further discussed. In fact, a few phenomena could have interfered with the blood parameters, including menstruations. However, since we had a male-dominated cohort with female patients on average over the age of menopause, the effect of bleeding (potentially leading to anemia) may have been limited in our cohort. Phenomena leading to more granulopoiesis, such as acute infections, also could have influenced blood parameters during treatment. Standard blood sample tests were performed at the same time as the FDG-PET/CT scans at all three time points (e.g., before treatment and three and six months after treatment start), we were so able to rule out major acute infections based on hybrid imaging performed before treatment and during the first six months of treatment. Furthermore, we also reported the onset of immune-related adverse events (IRAEs) during the first six months of treatment with ICIs, assuming that they might increase the lymphocyte counts. Interestingly, IRAEs were significantly more frequent in women, who had significantly lower counts of lymphocytes compared to men. These innovative findings will be extensively discussed in a separate manuscript currently under review. Finally, a detailed patient drug history should be documented to identify drugs with strong effect on granulopoiesis and erythrocyte and lymphocyte counts.

## 5. Conclusions

Inflammatory blood parameters predicted the short- and long-term responses to ICIs with a strong predictive power. Our results suggested the validation of inflammatory blood parameters as biomarkers that predict immunotherapies’ efficacity in metastatic melanoma patients. However, confounding factors that interfere with myelopoiesis should also be taken into consideration.

## Figures and Tables

**Table 1:** Patient characteristics before initiation of immune checkpoint inhibition.

**Median age at treatment start in years** **(IQR)**	68 (53–74)
**Sex**	***N* = 100**
*Male*	69
*Female*	31
**AJCC-stage at treatment start**	***N* = 100**
*III*	5
*IV*	95
**Histopathology of primary tumor, *n* (%)**	***N* = 100**
*Superficial spreading*	31
*Nodular*	27
*Lentigo maligna*	6
*Acral lentiginous*	8
*Sinonasal*	3
*Mucosal*	3
*Amelanotic*	1
*Ocular*	3
*Unknown*	18
**Site of primary tumor, *n* (%)**	***N* = 100**
*Head and Neck*	40
*Upper extremity*	22
*Body trunk*	14
*Lower extremity*	1
*Eye*	2
*Vagina*	1
*Sinonasal*	11
*No primary tumor*	9
**Site of melanoma metastases at treatment start, *n* (%)**	***N* = 670**
*Soft tissue*	359
*Lung*	142
*Liver/Spleen*	125
*Bone*	42
*Heart*	2
**Number of patients with one metastasis and *n* metastases at treatment start**	***N* = 100**
*1*	21
*2*	10
*3*	11
*4*	6
*5*	4
*6–10*	30
*11–15*	13
*>15*	5
**Prior treatment, *n* (%)**	***N* = 100**
*Native*	24
*Pretreated*	76
*Ipilimumab*	46
*Nivolumab*	13
*Ipilimumab + Nivolumab*	4
*BRAF Inhibitor*	3
*MEK Inhibitor*	4
*Chemotherapy*	4
*Intratumoral*	2
**Checkpoint inhibition, *n* (%)**	***N* = 100**
*Single*	84
*Double*	16

**Table 2:** Descriptive statistics of blood parameters in patients with disease control after six months of ICIs at baseline (TP 0), three months (TP 1), and six months (TP 2).

Characteristics	Minimum	1st Quantile	Median	Mean	3rd Quantile	Maximum
TP 0						
Basophiles (in g/L)	0.01	0.02	0.03	0.03	0.04	0.10
C-reactive protein (in mg/L)	0.30	1.20	3.90	17.46	11.75	310.00
Erythrocytes (per pL)	2.91	4.22	4.64	4.57	4.98	5.71
Leucocytes (in g/L)	3.02	5.35	6.54	7.07	7.99	16.28
Lymphocytes (in g/L)	0.21	1.08	1.35	1.45	1.67	4.60
Monocytes (in g/L)	0.19	0.40	0.54	0.59	0.74	1.40
Neutrophils (in g/L)	1.44	3.32	4.36	4.81	5.59	14.10
Thrombocytes (in g/L)	122.00	195.25	230.50	239.82	283.75	449.00
NLR	1.04	2.16	2.95	4.54	4.41	66.19
TP 1						
Basophiles (in g/L)	0.00	0.02	0.03	0.03	0.04	0.11
C-reactive protein (in mg/L)	0.30	1.00	2.95	9.63	6.45	137.00
Erythrocytes (per pL)	3.21	4.25	4.63	4.63	5.00	6.64
Leucocytes (in g/L)	3.53	5.72	6.62	6.98	8.45	11.79
Lymphocytes (in g/L)	0.60	1.11	1.44	1.60	1.90	5.68
Monocytes (in g/L)	0.15	0.40	0.55	0.57	0.69	1.60
Neutrophils (in g/L)	1.93	3.40	4.21	4.58	5.43	9.64
Thrombocytes (in g/L)	130.00	199.25	225.00	233.41	269.00	416.00
NLR	0.54	2.16	2.89	3.49	4.14	9.28
TP 2						
Basophiles (in g/L)	0.01	0.02	0.03	0.03	0.04	0.14
C-reactive protein (in mg/L)	0.30	1.10	2.40	6.71	4.70	151.00
Erythrocytes (per pL)	3.16	4.35	4.63	4.64	5.01	6.46
Leucocytes (in g/L)	3.24	5.29	6.56	6.79	8.55	12. 17
Lymphocytes (in g/L)	0.65	1.12	1.35	1.56	1.74	7.38
Monocytes (in g/L)	0.21	0.41	0.53	0.56	0.72	1.41
Neutrophils (in g/L)	1.55	3.06	4.25	4.39	5.54	9.44
Thrombocytes (in g/L)	118.00	201.50	238.50	233.65	264.00	449.00
NLR	0.584	1.93	2.75	3.37	4.07	9.25

**Table 3:** Descriptive statistics of blood parameters in patients with progressive disease after six months of ICIs at baseline (TP 0), three months (TP 1), and six months (TP 2).

Characteristics	Minimum	1st Quantile	Median	Mean	3rd Quantile	Maximum
TP 0						
Basophiles (in g/L)	0.01	0.02	0.03	0.03	0.05	0.07
C-reactive protein (in mg/L)	0.40	1.35	3.45	24.83	27.25	2 14.00
Erythrocytes (per pL)	2.87	3.92	4.25	4.23	4.75	5.19
Leucocytes (in g/L)	0.65	4.60	5.87	6.26	6.78	18.47
Lymphocytes (in g/L)	0.58	0.88	l.31	l.39	l.96	2.43
Monocytes (in g/L)	0.01	0.40	0.43	0.51	0.62	1.15
Neutrophils (in g/L)	0.01	2.60	3.69	3.52	4.31	6.59
Thrombocytes (in g/L)	121.00	202.50	219.00	247.04	273.25	473.00
NLR	0.02	1.73	2.54	2.71	3.27	5.89
TP l						
Basophiles (in g/L)	0.01	0.01	0.03	0.04	0.05	0.12
C-reactive protein (in mg/L)	0.40	2.32	5.95	17.63	31.00	102.00
Erythrocytes (per pL)	2.77	3.90	4.12	4.11	4.54	4.85
Leucocytes (in g/L)	4.78	5.27	6.59	6.89	8.15	l l.24
Lymphocytes (in g/L)	0.66	1.01	1.30	l.42	l.88	2.43
Monocytes (in g/L)	0.38	0.45	0.60	0.60	0.67	1.20
Neutrophils (in g/L)	1.92	3.11	4.50	4.55	5.57	9.39
Thrombocytes (in g/L)	150.00	221.25	266.00	279.27	325.75	534.00
NLR	1.11	2.49	3.17	3.63	4.21	8.45
TP 2						
Basophiles (in g/L)	0.01	0.01	0.02	0.03	0.04	0.11
C-reactive protein (in mg/L)	0.30	3.35	23.20	64.40	120.50	252.00
Erythrocytes (per pL)	l.99	3.50	4.03	3.91	4.35	4.90
Leucocytes (in g/L)	3.45	5.94	6.85	7.63	8.25	20.11
Lymphocytes(in g/L)	0.38	0.64	1.25	1.24	1.74	2.58
Monocytes (in g/L)	0.15	0.41	0.48	0.55	0.63	1.52
Neutrophy ls (in g/L)	2.01	3.54	4.56	5.60	6.16	18.66
Thrombocytes (in g/L)	147.00	227.00	251.00	270.04	302.50	429.00
NLR	1.12	2.62	4.85	6.04	9.03	20.97

## Data Availability

All reviewed imaging modalities and clinical data were assessed during clinical routine. Patient data are stored in local archiving system.

## Abbreviations

The following abbreviations were used in this manuscript:

CTLA-4	Cytotoxic T-lymphocyte-associated antigen-4 (CTLA-4)
PD-1	Programmed cell death protein-1 (PD-1)
PD-L1	Programmed cell death ligand-1
ICI	Immune checkpoint inhibitor
LDH	Lactate dehydrogenase
TMB	Tumor mutation burden
GCP	Good Clinical Practice
AJCC	American Joint Committee on Cancer
FDG-PET/CT	^18^F-2-fluor-2-desoxy-D-glucose positron emission tomography/computed tomography
TP	Time point
NLR	Neutrophils-to-lymphocytes ratio
TOF	Time-of-flight
BMI	Body mass index
RECIST	Response Evaluation Criteria in Solid Tumors
CR	Complete response
PR	Partial response
PD	Progressive disease
SD	Stable disease
OS	Overall survival
PFS	Progression-free survival
ROC	Receiver operating characteristic
IQR	Interquartile range
AIC	Akaike information criterion
CI	Confidence interval
